# A Working Framework to Address Diversity, Equity, and Inclusion in Undergraduate Medical Education

**DOI:** 10.1007/s40670-024-02065-1

**Published:** 2024-05-28

**Authors:** K. Jiang, J. B. Blumer, N. T. Zaveri, S. D. Schneid, E. M. Lee, J. L. Szarek, M. Kruidering, K. M. Quesnelle, M. W. Lee

**Affiliations:** 1 Department of Medical Education, Geisel School of Medicine, Dartmouth College, Remsen 232, 66 College Street, Hanover, USA; 2 Department of Cell and Molecular Pharmacology and Experimental Therapeutics, Medical University of South Carolina, Charleston, USA; 3 Department of Pharmacology, Arkansas Colleges of Health Education, Fort Smith, USA; 4 Skaggs School of Pharmacy and Pharmaceutical Sciences & School of Medicine, University of California San Diego, San Diego, USA; 5 Department of Medical Education, Geisinger Commonwealth School of Medicine, Scranton, USA; 6 Department of Cellular & Molecular Pharmacology, School of Medicine, University of California, San Francisco, San Francisco, USA; 7 Department of Biomedical Sciences, University of South Carolina School of Medicine Greenville, Greenville, USA; 8 Department of Family Medicine, Geisel School of Medicine, Dartmouth College, Hanover, USA

**Keywords:** Diversity, Inclusion, Gender, Medical education, PhIG

## Abstract

Health disparities exist among groups that are based on race, ethnicity, gender, socioeconomic status, and geography. Often, interventions directed at addressing these disparities are episodically incorporated into health professions education as opposed to a more uniform integration throughout a curriculum. Thus, a working framework for integrating and assessing diversity, equity, and inclusion (DEI) specifically into foundational science teaching in health professions’ education is needed. Current frameworks are theoretically based and often bereft of practical examples that basic science and clinical educators would find useful in educational settings. Here we analyzed examples in pharmacology, therapeutics, and clinical medicine to create a tool aimed at identifying and remediating biases and disparities across the undergraduate medical education (UME) curriculum. We initially focused on pharmacology examples and performed a literature search followed by an in-depth analysis of the literature together with our experiences teaching topics with a DEI component. It became clear that, in addition to pure pharmacology topics, there are many pharmacology- and therapeutics-related topics that also involve race, gender, and sexual orientation. These include clinical guidelines and clinical screening criteria. Further analysis of all of the examples derived from our multi-faceted analysis revealed common themes that we, in turn, compiled into a framework. This framework can be used by foundational science and clinical educators to help both students and faculty understand how to navigate DEI-associated foundational science content.

## Introduction

In response to social and cultural headwinds driven in part by tragic deaths, political turmoil, and the COVID-19 pandemic, there is a profoundly renewed focus on ensuring diversity, equity, and inclusion (DEI) in all areas of society, including the medical profession [1]. This has had a particularly powerful impact on students, classrooms, and educational venues, which has led to a myriad of efforts to incorporate DEI in education [2]. Consequently, a number of approaches and strategies have been defined that can help faculty practice inclusion, build trust, and improve participation from all students.

Indeed, the types of pedagogical approaches used in the classroom, the level of structure in the course, and instructor attitudes (i.e., empathy, supportiveness, self-awareness, knowledge about stereotype threat, cultural humility, etc.) can all influence the degree to which a classroom is perceived as inclusive [3–7]. Adoption of these approaches does not necessarily endorse an instructor as an expert on race, gender, or sexual orientation; rather it lets students know that instructors care about all learners’ engagement which, in turn, may create an environment of educational safety and improve learner outcomes [8]. For instance, active learning-based pedagogies that intentionally incorporate inclusive teaching have a superior impact on performance gaps compared to corresponding lecture-based sessions that lack an active learning component [5, 9]. Effective inclusive teaching practices that integrate relevant, culturally diverse examples promote access and equity for all students [7].

Within medical education and health science communities, professional societies have recognized the need to address inclusion and diversity as part of their mission and vision statements. Recently, the British Pharmacological Society codified their vision for DEI in five key principles that will help educators and researchers develop and deliver pharmacology programs [10]. Likewise, the Association of American Medical Colleges, in a recent press release, stated that “As leaders of anchor institutions in our communities, academic medicine’s physicians, educators, hospital leaders, faculty, researchers, learners, and staff must lead by example and take bold action in partnership with the communities we serve: We must take the lead in educating ourselves and others to address these issues head-on” [11]. Although these statements are a critical first step, they do not provide the level of detail that practitioners, researchers, and educators need to implement change and directly address issues in a serious and thoughtful manner. Furthermore, while institutions provide faculty development in addressing DEI, it is often generalized and may lack consistency within and among institutions.

More recently, the International Association of Medical Science Educators (IAMSE) published a manual (“Best Practices for Acknowledging and Addressing Racial and Ethnic Health Disparities in Medical Education”) and a toolkit (Diversity, Equity, and Inclusion Assessment Toolkit), both of which provide high-level strategies for addressing race and ethnicity in medical education [12]. However, there is a compelling need for specific, detailed examples that can be used in educational venues by educators to open the door for discussion and provide insight on why race, gender, or sexual orientation is, or is not, part of therapeutic guidelines or pharmacological treatment approaches. Thus, we designed a working framework to help educators and learners systematically navigate the complexities of race, gender, and culture in medicine that operates at the border of comfort, knowledge, and deeper understanding.

## Methods

### Literature Survey for DEI Practices in Pharmacology Education

We performed a PubMed search in 2022. We identified 266 papers using the following search strategy which included keywords/phrases and MeSH terms and no date limit:

(“DEI”[Title/Abstract] OR “diversity”[Title/Abstract] OR “equity”[Title/Abstract] OR “inclusion”[Title/Abstract] OR “race”[Title/Abstract] OR “Black”[Title/Abstract] OR “people of color”[Title/Abstract] OR “Latin*”[Title/Abstract] OR “ethnicity”[Title/Abstract] OR “sex”[Title/Abstract] OR “gender”[Title/Abstract] OR “bias”[Title/Abstract] OR “discrimination”[Title/Abstract] OR “racism”[Title/Abstract] OR “religion”[Title/Abstract] OR “nationality”[Title/Abstract] OR “socioeconomic”[Title/Abstract] OR “social”[Title/Abstract]) AND “Pharmacology”[MeSH Terms] AND (“Education”[Title/Abstract] OR “Curriculum”[Title/Abstract] OR “teach*”[Title/Abstract] OR “school*”[Title/Abstract] OR “universit*”[Title/Abstract]) Filters: English.

Of the 266 papers identified, we excluded those that were not directed toward education and did not address DEI. Based on this exclusion criterion, we reviewed 57 papers describing issues relevant to DEI and pharmacology. We further excluded studies with a pure research focus and were left with 11 papers that contained descriptions of DEI relevant to pharmacology education (e.g., clinical, medical, health sciences education).

### Identification and Discussion of Pharmacology and Therapeutic Content Related to DEI

**Fig. 1:**
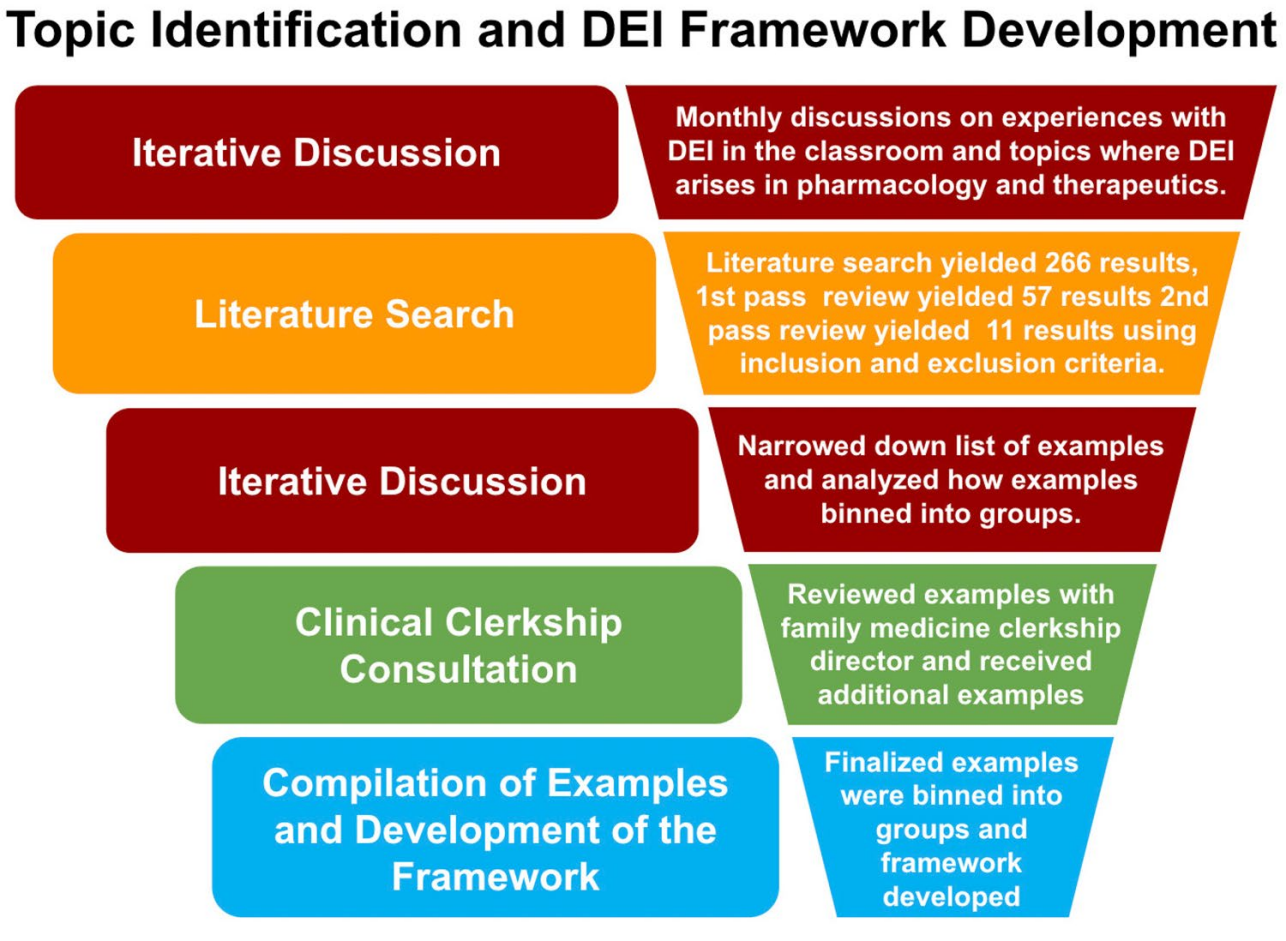
Diagram depicting authors’ workflow and specific outcomes at each step resulting in the final framework described in this manuscript

**Fig. 2:**
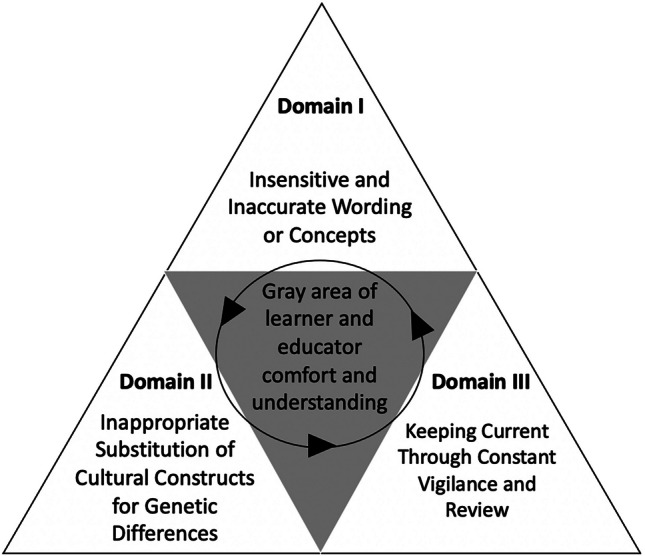
A framework for addressing DEI issues in UME curricula. The three domains of the framework inform each other and interact in a way to help educators and learners navigate the “gray area” of personal comfort and knowledge on DEI issues by promoting curiosity, discussion, and acknowledgement

Informed by the results of our literature search analysis, seven of the authors (JBB, NTZ, SDS, JLS, MK, KMQ, and MWL) had iterative discussions over the course of 24 months regarding their experiences teaching basic and clinical pharmacology including individual pharmacological agents, adverse effects, pharmacokinetics (absorption, distribution, metabolism, elimination), pharmacogenomics, therapeutic uses, as well as treatment and diagnostic guidelines, factors affecting medication adherence, therapeutic outcomes, gender pharmacology, and pharmacology assessment practices [13]. The issues raised in our discussions were further refined based on feedback received as we presented this work at numerous national and international poster and symposium sessions [14, 15]. Additional input was received from KJ, who is a third-year medical student at the Geisel School of Medicine at Dartmouth and clinical perspective was provided by EML, who is the Family Medicine clerkship director at Geisel School of Medicine at Dartmouth (Fig. 1). This was done to ensure the continuity of DEI considerations spanning the UME foundational sciences into learners’ clinical experiences and identified examples of DEI in medical practice. As a result of the authors discussions, a number of key topics and examples were identified where changes could be made to improve inclusion. Analysis of the different examples and topics revealed predominant themes that allowed grouping of the examples according to the conceptual framework articulated in Fig. 2. The framework is rooted in the principle of continuous improvement since these issues are continuing to evolve over time. Therefore, the purpose of this framework is to help all UME faculty review and address DEI issues in teaching in a generalizable manner, regardless of the discipline.

## Results

Based on our literature search analysis, iterative discussions, and analyses of UME curricular content taught at our institutions, we discovered two principal themes across the basic sciences and clerkships related to DEI: (1) topics and terminology/language that can be readily fixed, adjusted, or replaced to enhance inclusion and (2) topics that are more complex which require more thoughtful discussion with students. For example, some topics may require a discussion on the pros and cons of including race, sex, gender, or sexual orientation as part of medication management or guidelines. These discussions could be paired with didactic sessions on the guideline’s origins (i.e., the history of race in medicine). However, in some instances, topics may need to be addressed by an expert or be considered as part of a case discussion to allow time for deeper engagement (which is often limited in foundational science presentations). Regardless, there are multiple avenues via which DEI topics can be incorporated across medical curricula.

To conduct a systematic analysis of topics for embedding DEI in curricula, we formulated a framework which is divided into three different domains (Fig. 2). The first domain is focused on insensitive and inaccurate wording and concepts. The second domain concerns the inappropriate substitution of cultural constructs for genetic differences. Lastly, the third domain entails keeping curricula current through constant vigilance and review. The three domains operate in a cyclical manner linked by the “gray area of learner and educator comfort and understanding” which represents a region of interaction where significant benefit can be derived by discussing topics that are more complex and nuanced, not lending themselves to a single “right or wrong” answer. The goal of this framework is to foster open discussion to imbue educators and learners with the principle that this is a continuous cycle of analysis and improvement, not just a single pass of material.

### Framework for Systematic Analysis of DEI Issues in Undergraduate Medical Education Basic Science Content

#### Domain I. Insensitive and Inaccurate Wording or Concepts

Careful re-examination of teaching materials and content can ensure that the language used is not insensitive or inaccurate and that wording or concepts are free of bias. Removing or modifying harmful language or concepts may be under the control of the educator whereas in other cases we rely on outside societies or organizations to address these changes. Provided below are five clinically relevant DEI examples on the use of insensitive and inaccurate wording or concepts.

##### Example 1. Out-of-Date Guidelines That Still Include Race as a Variable

There are still a number of clinical guidelines used both in practice and teaching that continue to incorporate race as a variable (e.g., Joint National Committee (JNC) on Hypertension 8 guidelines) without compelling data to support its inclusion [16, 17]. The JNC 8 guidelines have not yet been updated to remove race as a factor, nor have the more recently developed 2017 American College of Cardiology/American Heart Association (ACC/AHA) guidelines, which also contain race as a variable [18]. However, these guidelines provide a remarkable opportunity to discuss study design, race, and treatment response.

##### Example 2. Potential for Harm by Removing Race as a Variable from Clinical Calculators

In contrast to JNC 8 or 2017 ACC/AHA guidelines, certain guidelines that have removed race may actually be harmful to certain vulnerable patient populations. For example, the MDRD (Modification of Diet in Renal Disease) formula for calculating glomerular filtration rate (GFR) was updated to remove race because race is not a biological construct, it does not adequately capture multi-racial diversity, and the original studies lacked sufficient evidence to support the inclusion of race [19]. However, removing race from the GFR formula may have a negative impact for self-identified Black/African American patients with both chronic kidney disease (CKD) *and* type 2 diabetes as eligibility and dosage of diabetes medication for these patients are determined by GFR [20]. This discordance can lead to confusion and frustration on the part of learners in the absence of sufficient explanation prior to their presentation and discussion. To help address this confusion, having a case conference or integrated session that includes professionals in primary care, specialists in kidney disease, and diversity experts can give learners a broad view of the issue both as it relates to medicine and DEI.

##### Example 3. Inaccuracies in Outdated Learning Materials

Older textbooks, primary literature, and secondary literature may contain language or references that may also contain insensitive language or biased references. For example, some older literature on acquired immunodeficiency syndrome (AIDS) still inappropriately incorporates the idea of a homosexual Canadian flight attendant named Gaeten Dugas as “patient zero” [21]. Educators using dated texts and literature with references to “patient zero” should strongly consider including a brief historical summary together with the lessons learned. This change would bolster the integration of both the epidemiology behind AIDS research as well as the sociocultural aspects that are often ignored or awkwardly tacked on to content.

Similarly, textbooks and literature provided to learners should be carefully reviewed for insensitive or inappropriate material. In 2017, the academic textbook publisher Pearson issued a recall and public apology for racially biased descriptions of “cultural differences in pain perception” found in a nursing text book (Nursing: A Concept-Based Approach to Learning, Volume I, Edition 2, 2014) [22].

##### Example 4. Updating Insensitive Language and Terms

For many years, the terms “red man syndrome” or “red neck syndrome” have been used to describe the adverse effect of the antibiotic vancomycin encountered in some patients upon infusion [23–25]. This term has the potential to marginalize certain groups of people. The potential for harm from using this term far outweighs the small effort it takes to proactively change or remove it from curricular materials.

##### Example 5. Addressing Stereotype and Bias During the Learning Process

Clinical vignettes may potentially reinforce stereotypes, introduce bias, and trigger stereotype threat, thereby hindering an examinee’s ability to perform well [26, 27]. Content that can be perceived as promoting stereotypes, bias, shame, or stigma spans many factors such as race, ethnicity, sex, gender, weight, religion, poverty, and disability, among others. Including these dimensions in vignettes can create negative stereotypes, even though exposing learners to the diversity of patients they will encounter in clinical practice is important. Examples include referring to a patient as homeless, rather than “a person experiencing homelessness” or referring to a patient as a “drug addict” rather than “a patient suffering from substance use disorder.” This issue requires careful use of language, prudence, and humility on the part of both educators and learners. However, this does not mean that all identifiers should be removed from vignettes. Rather, the key is to expose learners to a variety of patients without reinforcing stereotypes.

#### Domain II. Inappropriate Substitution of Cultural Constructs for Genetic Differences

There are many notable examples of using genetics to guide diagnosis, management, prognosis, and prevention in medical care. These include inherited diseases (e.g., cystic fibrosis or Huntington’s disease), drug therapy (e.g., certain anti-coagulants and anti-viral agents), disease progression (e.g., a range of cancer subtypes), and screening (e.g., breast cancer risk, congenital). While these examples rely on genetic differences between patients that may appear to align with race, ethnicity, or cultural ancestry, these associations are tenuous at best. As a consequence, there is a history of using race as a surrogate or proxy for genetics that can have harmful effects on patients and their care [28, 29]. There are now widespread efforts to review and revise the use of cultural constructs in medicine particularly in the context of genetic predisposition to disease or response to therapy.

Yet, in some instances where a clear understanding of genetic linkage is absent, race is still incorporated in screening, diagnosis, and management (e.g., prostate cancer, triple-negative breast cancer). These gray areas are the most difficult for educators and learners to navigate, though they furnish us with opportunities for deeper consideration of social constructs, genetics, and medicine. Examples of such gray areas can be found throughout the continuum of medical care.

Listed below are three examples that serve as prime opportunities to discuss the intersection of race, ethnicity, and genetics, and how this can directly impact a patient’s medical management outcome.

##### Example 1. Use of Ancestry in Cancer Screening

Prostate cancer screening is an example where it is recognized that self-identified Black/African American patients have a higher risk for developing prostate cancer compared to people of other races [30]. Thus, the American Urological Association (AUA) and the Society of Urologic Oncology (SUO) joint guidelines on PSA screening still include Black ancestry as a parameter and recommends screening at an earlier age than other populations. The current AUA/SUO guidelines state that:

“Clinicians should offer prostate cancer screening beginning at age 40 to 45 years for people at increased risk of developing prostate cancer based on the following factors: Black ancestry, germline mutations, strong family history of prostate cancer. (*Strong Recommendation; Evidence Level: Grade B*)” [31].

At some point in time, there may be a biological or genetic marker that helps explain this increased incidence. However, at the current time, Black ancestry is still part of the guidelines. If learners were simply provided such guidelines without explanation or discussion (i.e., inclusion of race may benefit some patients in certain situations), then this can lead to stress and discomfort and a perception that no effort was taken to address potential bias. Yet, the guidelines and position statement by the AUA/SUO is actually instructive for teaching and learning efforts. The AUA guidelines have been updated to include a position statement and acknowledge the risks of including race: “*Although emerging data exist, a far more comprehensive understanding is required of the impact of race and ethnicity on the operating characteristics of PSA, secondary biomarkers, and prostate imaging. It is also essential to recognize many people undergoing screening are of mixed (or unknown) race and ethnicity. Since dramatic disparities exist regarding access and affordability of certain diagnostic or imaging modalities, efforts should be made by clinicians, payors, and health care systems to bridge this gap*.” This statement, in and of itself, provides a learning opportunity regarding recognition and acknowledgment of DEI in medical practice.

##### Example 2. Focusing on Disease Prevalence in Racial Groups Can Lead to Harmful Generalizations

Triple-negative breast cancer (TNBC) more commonly occurs in self-identified Black/African American women compared to people of other races and genders (i.e., TNBC in males, while rare, can occur) [32]. This widely reported observation, while accurate, can lead to the harmful assumption that TNBC only affects self-identified Black/African American women or is strictly associated with race and gender. Taking into consideration that both genetic (biological) and non-genetic (lifestyle) factors appear to play a role in pathogenesis makes TNBC an ideal vehicle to discuss the intersection of cultural constructs and genetics [33, 34].

##### Example 3. Genetics and Adverse Drug Reactions

The adverse drug reaction known as Stevens-Johnson syndrome (SJS) is associated with some types of medications (e.g., agents for management of gout, anti-convulsant drugs) in patients with mutations in certain human leukocyte antigen (HLA) genes [35]. Some HLA polymorphism–associated adverse drug reactions are disproportionately represented in patients who have self-identified as certain ethnicities, such as Black, Asian, and Indian [36, 37]. However, while SJS develops more commonly in these ethnic groups after treatment with certain medications, it does not *only* occur in these ethnicities. In the absence of a better understanding of the role that complex population genetics play in this adverse drug reaction, teachers, learners, and practicing physicians are left with an incomplete understanding that exposes patients to both potential bias and serious medical complications. Articulating that prevalence is not a blanket permission to overlay race, ethnicity, and genetics is important for learners to hear, given that mixed ancestry as well as unknowns about biology can render the relationship between race and genetics invalid.

#### Domain III. Keeping Current Through Constant Vigilance and Review

Our understanding of the intersection of medicine, genetics, race, ethnicity, sexual orientation, and gender is constantly evolving. As this relationship develops and new knowledge is generated, educators and learners must stay up to date on changes. Additionally, as our understanding changes, so too does the language we use, which has the potential to promote or propagate bias. Thus, it is imperative that educators and learners stay current and up to date on changes in the field of DEI. Likewise, educators and learners need to consider the ever changing political, religious, and cultural influences that can shape medical education and presentation of certain topics (e.g., abortion, transgender care). These influences often vary by region, state, or even among local hospital systems. Provided below are two examples where constant vigilance and review are warranted.

##### Example 1. Continual Learning of Transgender Health

Current medical care and terminology relating to transgender patients provides a cautionary tale for those who believe that complacency in addressing DEI issues in the classroom is acceptable. While transgender patients have always been a part of our society and medical practice, more visibility has been garnered through advocacy. As a result, society and health science professions grapple with both biological issues and their role in management, diagnosis, and patient counseling. Terminology and proper use of pronouns is evolving. A growing body of research continues to highlight health inequities experienced by transgender individuals, and further underscores the need for medical providers to be appropriately trained to deliver care to this population [38, 39]. A number of tools have been developed and reported in the literature to address gender and transgender health inequities in education and clinical care [40, 41]. These resources can be utilized to improve attitudes and awareness toward transgender populations, provide knowledge of unique clinical concerns, and help develop skills to deliver competent care [40].

Research suggests that incorporating transgender clinical competencies across the curriculum (rather than in one or two condensed lectures) helps reinforce topics in their relevant clinical context with emphasis over time [39, 42]. Because of rapid changes in our society, cases and material should be reviewed regularly and updated. For example, the aforementioned AUA/SUO PSA prostate cancer screening guidelines also acknowledge gender as a potential source of bias: “*For non-binary patients or transgender women there is a lack of data on prostate cancer screening preferences, if and when to initiate, the accuracy of biomarkers (e.g., PSA, secondary biomarkers, MRI), potential psychological consequences, impact of gender-affirming hormonal therapy, and priorities regarding management options. Considerably more effort and research are required*.” [31]. This facet of patient care may escape some learners and educators who are only thinking about male patients in regard to prostate cancer. This could lead to a lack of consideration for a pathological issue that could affect male patients who have transitioned to female. This highlights the importance of carefully reviewing resources for changes so that teaching material can be updated appropriately.

##### Example 2. Impact of Gender Bias

Gender bias represents another area of clinical medicine which has unique challenges [43]. For example, gender bias has permeated published textbooks used in medical education [44]. While strides have been made to address bias within some areas of gender, the coverage of topics specifically highlighting transgender populations is often inadequate or altogether missing in medical and health science curricula. The lack of knowledge surrounding transgender health has manifested in reports of transgender people being denied health care or experiencing discrimination, including verbal and physical abuse, even in health care settings [45, 46].

## Discussion

There is an imperative need in medical education to make learning inclusive so that all students feel involved, engaged, welcomed, and that no one feels marginalized. A key part of this process is helping learners identify their own personal biases and implicit biases [47]. Another critical skill is teaching learners how to *assess materials* for potential bias.

To help address these challenges, we designed a framework for health science educators to structure efforts to review curricular content for language and practices that could marginalize underrepresented populations. Moreover, this framework goes beyond the pharmacology examples we promulgated and extends into many related areas of foundational and clinical undergraduate medical education. Indeed, this framework can help educators and students become directly engaged in ensuring DEI is addressed in basic science content by promoting careful reflection and discussion of difficult issues. This framework consists of three domains, covering the inappropriate use of wording and concepts, race as a social construct, and constant vigilance, combined with humility.

When considering DEI in an education setting, there may be a temptation to either tack on examples to existing material, which simply checks a box, or to avoid discussion altogether. Neither of these approaches help address the problems at hand and only serve to exacerbate them. Difficult and complicated scenarios where cultural constructs collide with medicine, which live in a gray area, provide us with phenomenal opportunities to teach skills that will help learners grow into compassionate and inclusive practitioners. Carefully sourcing material and scenarios that allow educators and learners to view the issues from multiple perspectives can lead to revelations that drive changes in personal behavior, such as reducing implicit bias [48].

Race, gender, and sexual orientation frequently appear as co-variates in literature, where it could lead educators, learners, and health care professionals to make erroneous assumptions. A recent paper on germline genetic screening for patients with a cancer diagnosis illustrates how we can stimulate objective discussion while paying heed to DEI. For example, Kurian et al. highlight how the prevalence of genetic screening following cancer diagnosis is lower among patients who identify as Asian, Black/African American, and Hispanic for cancers that have the highest overall screening rates (e.g., male breast cancer, female breast cancer, and ovarian cancer) [49]. The way that a patient’s race was “documented” and the relationship of genetic penetrance to phenotype are both valid points with any study of this nature, and these should be discussed.

Yet, the issue here is not that cultural constructs are being used inappropriately or as a surrogate for genetics of disease (all of the patients in the study had cancer) but rather as an index to reveal which populations of patients are under-screened. This means that the rates of screening (not cancer rates, underlying patient biology/genetics) vary among different groups identified by race/ethnicity. Thus, the issue is not about differences in patients’ genetics but about *access* to patients’ genetics, which may by hindered by patients *access* to health care, *access* to insurance, or *access* to knowledge regarding the importance of screening. Applying the framework described in this paper can help educators and learners carefully consider how to best help patients in a way that does not harm or exclude them without compromising their medical care.

All of these efforts, however, only work effectively if there is support from leadership, support to implement frameworks, support to create time for discussions, and support to share resources. Invariably when educators or clinicians present material to learners, whether DEI-related or not, there is a possibility that learners can be triggered and react negatively. If this occurs in a learning environment, then the instructor has the opportunity to address the issue and shape the experience into a learning event. If, however, the learners do not share the information during the session and it escalates to academic leadership directly or emerges in a delayed fashion, such as post-course evaluations, it can have disastrous consequences for all parties involved and it risks reversing efforts to engage in discussion. Thus, using this framework to identify and triage DEI issues is only the first step. Implementing strategies for reducing implicit bias in tandem with examples identified using the framework stands to actually help educators and learners become less biased and more engaged in productive conversations [50].

## Conclusion

The framework outlined here is highly generalizable across health science professions and basic/foundational sciences that discuss the impact of genetic differences across different populations. Using this framework, educators can effectively incorporate DEI in curricula and, with long-term use, this additional education may help reduce disparities in health care outcomes based on these social constructs. Future efforts should be dedicated to studying the impact of improving learner inclusion and incorporation of DEI on student performance and patient outcomes.

## References

[1] Vela MB, Chin MH, Peek ME. Keeping our promise - supporting trainees from groups that are underrepresented in medicine. N Engl J Med. 2021;385(6):487–9. 34329547 10.1056/NEJMp2105270 PMC8663282

[2] Anderson AN, Chan AR, Roman YM. Pharmacogenomics and clinical cultural competency: pathway to overcome the limitations of race. Pharmacogenomics. 2022;23(6):363–70. 35311348 10.2217/pgs-2022-0009

[3] Burgess DJ, Warren J, Phelan S, Dovidio J, van Ryn M. Stereotype threat and health disparities: what medical educators and future physicians need to know. J Gen Intern Med. 2010;25(2):S169-77. 20352514 10.1007/s11606-009-1221-4 PMC2847106

[4] Dewsbury B, Brame CJ. Inclusive teaching. CBE Life Sci Educ. 2019;18(2):fe2. 10.1187/cbe.19-01-0021 PMC7058128 31025917

[5] Dewsbury BM, Swanson HJ, Moseman-Valtierra S, Caulkins J. Inclusive and active pedagogies reduce academic outcome gaps and improve long-term performance. PLoS ONE. 2022;17(6): e0268620. 35704639 10.1371/journal.pone.0268620 PMC9200326

[6] Freeman S, Haak D, Wenderoth MP. Increased course structure improves performance in introductory biology. CBE Life Sci Educ. 2011;10(2):175–86. 21633066 10.1187/cbe.10-08-0105 PMC3105924

[7] Tanner KD. Structure matters: twenty-one teaching strategies to promote student engagement and cultivate classroom equity. CBE Life Sci Educ. 2013;12(3):322–31. 24006379 10.1187/cbe.13-06-0115 PMC3762997

[8] Williamson AJH, Jensen RM, Smith BK. Educational safety for the surgical learner. J Surg Educ. 2022;79(5):1083–7. 35525777 10.1016/j.jsurg.2022.04.007

[9] Theobald EJ, Hill MJ, Tran E, et al. Active learning narrows achievement gaps for underrepresented students in undergraduate science, technology, engineering, and math. Proc Natl Acad Sci U S A. 2020;117(12):6476–83. 32152114 10.1073/pnas.1916903117 PMC7104254

[10] British Pharmacological Society. Principles for inclusive implementation of the undergraduate pharmacology core curriculum. https://www.bps.ac.uk/education-engagement/teaching-pharmacology/undergraduate-curriculum/inclusive-principles. Accessed June 18th, 2023.

[11] Association of American Medical Colleges. AAMC Statement on Police Brutality and Racism in America and Their Impact on Health. https://www.aamc.org/professional-development/affinity-groups/cfas/cfas-societies-sign-aamc-statement-racism-and-police-brutality. Updated June 1^st^, 2020. Accessed June 15th, 2023.

[12] Powell J.M, Linger R. M. A. Best practices for acknowledging and addressing racial and ethnic health disparities in medical education (2023). Springer International Publishing.

[13] Quesnelle KM, Zaveri NT, Schneid SD, Blumer JB, Szarek JL, Kruidering M, Lee MW. The importance of collaboratively designing pharmacology education programs. Pharmacol Res Perspect. 2021;9(3): e00773. 33974347 10.1002/prp2.773 PMC8112302

[14] Blumer JB, Szarek JL, Lee MW, Zaveri N, Schneid SD, Kruidering M, Quesnelle KM. Addressing race, ethnicity, and structural inequality in pharmacology education and assessment. Medical Science Educator. 2022;32(1):23–123.

[15] Blumer JB, Szarek JL, Lee MW, et al. Addressing Race, ethnicity, and structural inequality in pharmacology education and assessment. The FASEB Journal. 2022;36(S1).

[16] Armstrong C, Joint NC. JNC8 guidelines for the management of hypertension in adults. Am Fam Physician. 2014;90(7):503–4. 25369633

[17] Westby A, Okah E, Ricco J. Race-based treatment decisions perpetuate structural racism. Am Fam Physician. 2020;102(3):136–7. 32735444

[18] Whelton PK, Carey RM, Aronow WS, et al. 2017 ACC/AHA/AAPA/ABC/ACPM/AGS/APhA/ASH/ASPC/NMA/PCNA guideline for the prevention, detection, evaluation, and management of high blood pressure in adults: executive summary: a report of the American College of Cardiology/American Heart Association Task Force on Clinical Practice Guidelines. J Am Coll Cardiol. 2018;71(19):2199–269. 29146533 10.1016/j.jacc.2017.11.005

[19] Organ Procurement and Transplantation Network. Understanding race and eGFR. Health Resources and Services Administration, U.S. Department of Health & Human Services. https://optn.transplant.hrsa.gov/patients/by-organ/kidney/understanding-the-proposal-to-require-race-neutral-egfr-calculations. Accessed September 13, 2023.

[20] Walther CP, Winkelmayer WC, Navaneethan SD. Black race coefficient in GFR estimation and diabetes medications in CKD: national estimates. J Am Soc Nephrol. 2021;32(6):1319–21. 33833077 10.1681/ASN.2020121724 PMC8259644

[21] McKay RA. “Patient zero”: the absence of a patient’s view of the early North American AIDS epidemic. Bull Hist Med. 2014;88(1):161–94. 24769806 10.1353/bhm.2014.0005 PMC4046389

[22] Jaschik S. Anger over stereotypes in textbook. Inside Higher Ed. October 22, 2017. https://www.insidehighered.com/news/2017/10/23/nursing-textbook-pulled-over-stereotypes. Accessed September 13th, 2023.

[23] Bradford A. “Red man syndrome”: thoughts on an anachronistic term. Fam Med. 2013;45:509–10. 23846972

[24] Alvarez-Arango S, Ogunwole SM, Sequist TD, Burk CM. Blumenthal KG. Vancomycin infusion reaction -moving beyond “red man syndrome.” N Engl J Med. 2021;384(14):1283–6. 33830710 10.1056/NEJMp2031891 PMC9235042

[25] Korman TM. Moving beyond “red man syndrome.” N Engl J Med. 2021;385(2):192. 34233103 10.1056/NEJMc2107779

[26] Steele CM. Thin ice: stereotype threat and Black college students,. *The Atlantic Monthly* August 1st, 1999. Retrieved from http://www.theatlantic.com/past/docs/issues/99aug/9908stereotype.htm Accessed September 13th, 2023.

[27] Beilock SL, Rydell RJ, McConnell AR. Stereotype threat and working memory: mechanisms, alleviation, and spillover. J Exp Psychol Gen. 2007;136(2):256–76. 17500650 10.1037/0096-3445.136.2.256

[28] Duello TM, Rivedal S, Wickland C, Weller A. Race and genetics versus ‘race’ in genetics: a systematic review of the use of African ancestry in genetic studies. Evol Med Public Health. 2021;9(1):232–45. 34815885 10.1093/emph/eoab018 PMC8604262

[29] Cerdena JP, Asabor EN, Plaisime MV, Hardeman RR. Race-based medicine in the point-of-care clinical resource UpToDate: a systematic content analysis. EClinicalMedicine. 2022;52: 101581. 35923427 10.1016/j.eclinm.2022.101581 PMC9340501

[30] Lillard JW Jr, Moses KA, Mahal BA, George DJ. Racial disparities in Black men with prostate cancer: a literature review. Cancer. 2022;128(21):3787–95. 36066378 10.1002/cncr.34433 PMC9826514

[31] Wei JT, Barocas D, Carlsson S, et al. Early detection of prostate cancer: AUA/SUO guideline Part I: Prostate cancer screening. J Urol. 2023;210(1):46–53. 37096582 10.1097/JU.0000000000003491 PMC11060750

[32] Brewster AM, Chavez-MacGregor M, Brown P. Epidemiology, biology, and treatment of triple-negative breast cancer in women of African ancestry. Lancet Oncol. 2014;15(13):e625–34. 25456381 10.1016/S1470-2045(14)70364-X PMC4413447

[33] Prakash O, Hossain F, Danos D, Lassak A, Scribner R, Miele L. Racial disparities in triple negative breast cancer: a review of the role of biologic and non-biologic factors. Front Public Health. 2020;8: 576964. 33415093 10.3389/fpubh.2020.576964 PMC7783321

[34] Chang CS, Kitamura E, Johnson J, Bollag R, Hawthorn L. Genomic analysis of racial differences in triple negative breast cancer. Genomics. 2019;111(6):1529–42. 30366040 10.1016/j.ygeno.2018.10.010

[35] Tangamornsuksan W, Chaiyakunapruk N, Somkrua R, Lohitnavy M, Tassaneeyakul W. Relationship between the HLA-B*1502 allele and carbamazepine-induced Stevens-Johnson syndrome and toxic epidermal necrolysis: a systematic review and meta-analysis. JAMA Dermatol. 2013;149(9):1025–32. 23884208 10.1001/jamadermatol.2013.4114

[36] Tangamornsuksan W, Chanprasert S, Nadee P, et al. HLA genotypes and cold medicine-induced Stevens-Johnson syndrome/toxic epidermal necrolysis with severe ocular complications: a systematic review and meta-analysis. Sci Rep. 2020;10(1):10589. 32601360 10.1038/s41598-020-67610-5 PMC7324363

[37] Lu N, Rai SK, Terkeltaub R, Kim SC, Menendez ME, Choi HK. Racial disparities in the risk of Stevens-Johnson syndrome and toxic epidermal necrolysis as urate-lowering drug adverse events in the United States. Semin Arthritis Rheum. 2016;46(2):253–8. 27217070 10.1016/j.semarthrit.2016.03.014 PMC5035554

[38] Institute of Medicine (US) Committee on Lesbian, Gay, Bisexual, and Transgender Health Issues and Research Gaps and Opportunities. The health of lesbian, gay, bisexual, and transgender people: building a foundation for better understanding. Washington (DC): National Academies Press (US); 2011. https://www.ncbi.nlm.nih.gov/books/NBK64806/. 22013611

[39] Dubin SN, Nolan IT, Streed CG Jr, Greene RE, Radix AE, Morrison SD. Transgender health care: improving medical students’ and residents’ training and awareness. Adv Med Educ Pract. 2018;9:377–91. 29849472 10.2147/AMEP.S147183 PMC5967378

[40] Coleman E, Radix AE, Bouman WP, et al. Standards of care for the health of transgender and gender diverse people, version 8. International Journal of Transgender Health. 2022;23(sup1):S1–259. 36238954 10.1080/26895269.2022.2100644 PMC9553112

[41] Weiss LB, Levison SP. Tools for integrating women’s health into medical education: clinical cases and concept mapping. Acad Med. 2000;75(11):1081–6. 11078666 10.1097/00001888-200011000-00012

[42] Ferrara E, Pugnaire MP, Jonassen JA, et al. Sexual health innovations in undergraduate medical education. Int J Impot Res. 2003;15(Suppl 5):S46-50. 14551577 10.1038/sj.ijir.3901072

[43] Mayer KH, Bradford JB, Makadon HJ, Stall R, Goldhammer H, Landers S. Sexual and gender minority health: what we know and what needs to be done. Am J Public Health. 2008;98(6):989–95. 18445789 10.2105/AJPH.2007.127811 PMC2377288

[44] Dijkstra AF, Verdonk P, Lagro-Janssen AL. Gender bias in medical textbooks: examples from coronary heart disease, depression, alcohol abuse and pharmacology. Med Educ. 2008;42(10):1021–8. 18761614 10.1111/j.1365-2923.2008.03150.x

[45] Association of American Medical Colleges. Implementing curricular and institutional climate changes to improve health care for individuals who are LGBT, gender nonconforming, or born with DSD. https://store.aamc.org/implementing-curricular-and-institutional-climate-changes-to-improve-health-care-for-individuals-who-are-lgbt-gender-nonconforming-or-born-with-dsd-a-resource-for-medical-educators.html. Updated 2014. Accessed June 15th, 2023.

[46] Grant JM, Mottet LA, Tanis J. National Transgender Discrimination Survey: full report. https://transequality.org/issues/resources/national-transgender-discrimination-survey-full-report. Updated September 11^th^, 2012. Accessed June 15th, 2023.

[47] Ruben M, Saks NS. Addressing implicit bias in first-year medical students: a longitudinal, multidisciplinary training program. Med Sci Educ. 2020;30(4):1419–26. 34457809 10.1007/s40670-020-01047-3 PMC8368581

[48] Galinsky AD, Moskowitz GB. Perspective-taking: decreasing stereotype expression, stereotype accessibility, and in-group favoritism. J Pers Soc Psychol. 2000;78(4):708–24. 10794375 10.1037//0022-3514.78.4.708

[49] Kurian AW, Abrahamse P, Furgal A, et al. Germline genetic testing after cancer diagnosis. JAMA. 2023;330(1):43–51. 37276540 10.1001/jama.2023.9526 PMC10242510

[50] Devine PG, Forscher PS, Austin AJ, Cox WT. Long-term reduction in implicit race bias: a prejudice habit-breaking intervention. J Exp Soc Psychol. 2012;48(6):1267–78. 23524616 10.1016/j.jesp.2012.06.003 PMC3603687

